# Transformational leadership and predictors of resilience among registered nurses: a cross-sectional survey in an underserved area

**DOI:** 10.1186/s12912-023-01192-1

**Published:** 2023-02-10

**Authors:** Hana’a Abdul Salam, Nuhad Yazbik Dumit, Michael Clinton, Ziyad Mahfoud

**Affiliations:** 1 Hariri School of Nursing, American University of Beirut, P.O. Box: 11-0236, Riad El Solh, Beirut, 1107 2020 Lebanon; 2 Weill Cornell Medicine-Qatar, P.O. Box: 24144, Doha, Qatar

**Keywords:** Cross sectional-survey, Leadership, Nurses, Psychological resilience

## Abstract

**Background:**

High resilience increases nurses' ability to cope with job-related stressors and enhances job satisfaction and, consequently, their retention. The study aims to identify resilience predictors and perceptions of transformational leadership in a convenience sample of registered nurses in Lebanon.

**Methods:**

An anonymous cross-sectional survey of a convenience sample of 240 registered nurses working for more than a year at three private hospitals in an underserved area in South Lebanon was used. The survey instrument included demographic questions, the True Resilience Scale ©, and the Global Transformational Leadership Scale. Multiple linear regression was used to assess the predictors of resilience after a descriptive analysis of the study variables.

**Results:**

The survey response rate was 85%. The nurses' mean resilience score was 119.4 (SD 15.3), and their perception of transformational leadership score was M = 25.0, SD = 6.8. Compared to bedside nurses, nurse managers, nurses with more than five years of experience, and nurses in critical nursing units had statistically significant higher resilience scores (*p* < 0.05). Resilience scores and perception of global transformational leadership were moderately correlated (*r* = 0.53, *p* < 0.05). In the final multiple linear regression model, 30% of the variation in resilience scores was explained by designation (*p* < 0.05) and perception of Global Transformational Leadership (*p* < 0.01). Perception of global transformational leadership scores explained 29% of the variance in resilience scores. Designation and perception of global transformational leadership predicted resilience in this sample.

**Conclusions:**

A national survey of the Lebanese nursing workforce is needed to achieve an improved predictive model and support policy developments to increase resilience among bedside nurses and retain them in the nursing workforce. Nurse administrators can help by strengthening their transformational leadership behaviors. Consistent use of transformational leadership styles will strengthen bedside nurses' resilience, increase nurse retention, and help sustain the Lebanese nursing workforce.

## Introduction

An increase in demand and a decrease in supply causes the global nursing shortage [1]. The State of the World's Nursing Report (2020) estimated a global nursing shortage of 5.9 million nurses in 2018, an improvement of 0.7 million nurses since 2016 [2]. In the United States, the estimated average cost of nurse turnover ranges from $44,000 to $64,000 per nurse [3]. In Lebanon, the cost of nurse turnover is twice the salary of a registered nurse, approximately $3,000 [4].

Lebanon has a shortage of qualified nurses, with 16.74 nurses and midwives per 10,000 population compared to an average of 24 nurses and midwives per 10,000 population in the Eastern Mediterranean region, thus ranking 10 of 21 countries in the region [5]. Gulf countries heavily recruit Lebanese nurses. The migration of nurses is a significant issue in Lebanon. Previous studies have shown that 20% of nurses leave the country within two years of graduation. The Order of Nurses in Lebanon records show that nurse migration is increasing [6]. Burmeister et al. (2019) reported that 43% of registered nurses working at a large university-affiliated hospital intended to migrate from Lebanon within 12 months [7]. Reasons for nurse migration from Lebanon include poor salaries and benefits, lack of professional development opportunities and career progression, unsupportive working environment, poor working conditions, lack of security, heavy workloads, absence of job stability, and moving with the family [6].

Healthcare professionals live amid many stressors including resource shortages, time constraints, emotional concerns [8], and trauma [9]. Nurses are challenged by lack of autonomy and low-level staff support [8], organizational changes, excessive workloads [10], violence [11], shift work, and insufficient resources [9]. These challenges are associated with retention problems [12]. Consequently, the remaining nurses suffer from heavy workloads, burnout, and job dissatisfaction [13]. The situation is especially grave in Sidon and its suburbs where this study took place; the economic situation is catastrophic, and the community lacks health and other services. Sidon has become a poverty zone [14]. Furthermore, healthcare in the city and its surroundings is compromised by the low ratio of nurses to the population, 1.7/100000 [15].

Sidon is the third largest city in Lebanon with a high concentration of the urban underprivileged. The city is suffering from poor essential services such as comprehensive healthcare services, education, electricity, and other needs associated with social tensions. Moreover, as the district of the south was separated into two parts in 1995, Sidon witnessed a decline in its administrative importance, particularly after the decentralization laws of municipalities [14]. Consequently, the flow of investments into greater Sidon was considerably reduced. The ratio of the urban poor is estimated to exceed twice that of the national poverty rate [14]. Sidon has one university-affiliated hospital and five other hospitals. One of the six hospitals is a government hospital; the other five are private hospitals [16]. In addition, there are five Ministry of Public Health-accredited primary healthcare centers [17].

In an environment where tragedy, human suffering, and distress are considered major components of nurses' daily lives, resilience emerges as an essential requirement of nurses’ survival [18]. Resilience is defined as "the ability of people or things to feel better quickly after something unpleasant, such as shock, injury, etc." [19]. It is the capability to survive life challenges, learn and grow from them, and become stronger. This capacity governs people's response to change [20]. Since resilient individuals are in a better position to deal with the stresses of workplace changes [21], resilient nurses are a vital part of a sustainable healthcare workforce [22]. Studies have shown that resilience is associated with employee job satisfaction and anticipated turnover [23], performance, organizational commitment, and ultimately organizational performance [21]. There is an apparent connection between perceived stress, job satisfaction, burnout, and organizational withdrawal, which leads to voluntary turnover [21]. Resilience can decrease voluntary turnover since it improves employee wellbeing and productivity [23]. Investigators have reported contradictory results about personal attributes as predictors of resilience [24].

Employee resilience is promoted by better psychological health, which is impacted by leadership style [25]. Transformational leaders encourage high performance by engaging with employees [26]. As a result, transformational leadership enhances followers' positive psychological capital [27]. In addition, transformational leadership is associated with employee empowerment and increased motivation [28]. Transformational leadership is positively correlated with worker engagement, organizational commitment, and job satisfaction [29]. Furthermore, transformational leadership predicts job satisfaction [30], which in turn predicts nurses' intent to stay [31].

Transformational leadership is a management practice; it is a leadership style that constitutes a set of leadership behaviors that inspire and empower followers to go beyond what is required and expected [25]. This type of leadership is characterized by its four dimensions, specifically idealized influence, inspirational motivation, intellectual stimulation, and individual consideration. Idealized influence occurs when leaders develop trust and respect among followers through exhibiting ethical behaviors. Inspirational motivation occurs when leaders inspire followers by communicating high expectations. Intellectual stimulation happens when followers are encouraged to see issues from different perspectives. Individual consideration occurs when leaders act upon followers' concerns and needs [25].

Transformational leadership has a positive impact on employees' psychological health and resilience [25]. Arnold (2017) found that psychological empowerment mediates the relationship between transformational leadership and employees' subjective wellbeing [32]. Moreover, Ehrnooth et al. (2021) reported a statistically significant relationship between transformational leadership and self-efficacy, a component of resilience [33]. Transformational leadership may predict higher levels of resilience among nurses because transformational leaders have higher motivation and are, therefore, more committed to their organizations than others [34].

Since high resilience increases nurses’ ability to cope with work stressors and increases their job satisfaction, it is vital to study nurses' resilience and perceptions of global transformational leadership. Similarly, it is crucial to explore how nurses in Sidon and other disadvantaged areas perceive transformational leadership and how their perceptions affect their resilience.

The scarcity of data regarding resilience and nurses' perceptions of transformational leadership in underserved areas of Lebanon needs investigation as the research findings might assist healthcare organizations in retaining nurses by increasing their resilience and improving their job satisfaction. Therefore, policymakers and nurse administrators might find the findings of this study helpful when developing strategies and implementing initiatives intended to create and sustain the nursing workforce in Lebanon's underserviced areas. Moreover, the findings of this study may inform other nurses’ and healthcare authorities in other countries having similar circumstances and serving similar communities affected by economic adversities.

To our knowledge, no studies in Lebanon or in the region have investigated the relationship between self-reported resilience among nurses and their perceptions of transformational leadership.

This study explores resilience among a convenience sample of hospital nurses working in Sidon, South Lebanon. A related aim is to identify the relationship of perceived global transformational leadership and nurse designation (bedside care vs. management role) on resilience in this sample of nurses working in a disadvantaged area.

The specific research questions are:

- 1. What are the perceived levels of resilience and perceptions of global transformational leadership of the registered nurses in the sample?
- 2. Do registered nurses with more positive perception of global transformational leadership have higher resilience scores?
- 3. Which study variables predict resilience among registered nurses in the sample?

## Material and methods

### Sample and setting

A quantitative cross-sectional descriptive study design using survey questionnaire was conducted to determine the average resilience score and predictors of resilience among registered nurses and their relationship to nurses' perception of global transformational leadership in Sidon in South Lebanon at three private hospitals in March 2016. The study data were collected from March 2^nd^ to March 22^nd^, 2016, after the American University of Beirut Institutional Review Board (IRB ID: NUR.ND.11) and hospital directors at the three hospitals approved the study. The hospital directors were assured that only the aggregated study results would be reported.

A convenience sample of 240 registered nurses who have been working at the three private hospitals for more than one year was included in the study. Practical nurses and auxiliaries were excluded from the study since the study aims to identify predictors and perceptions of transformational leadership in a convenience sample of registered nurses in Lebanon. The participants were 173 registered nurses and 30 nurse managers. Registered nurses are healthcare professionals licensed by the Ministry of Public Health to practice nursing. The nurse managers are registered nurses with managerial responsibilities 24 h a day, 7 days a week for a patient care unit. The sample distribution included 120 nurses from hospital A, 70 nurses from hospital B, and 50 nurses from hospital C depending on the number of registered nurses at each hospital. Nurses who met the inclusion criteria (registered nurses providing direct patient care and nurse managers) confirmed their voluntary consent on the first page of the survey. Informed consent was obtained from all subjects. Practical nurses and auxiliaries were excluded from the study.

### Instruments

The study questionnaire included demographic and work-related questions in addition to The Original Resilience Scale © [35] and the Global Transformational Leadership (GTL) Scale [36].

Demographic and work-related data fields included age, gender, marital status, number of dependents, nursing experience, educational level, designation (bedside nurse or nurse manager), and clinical specialty.

The original Resilience Scale © [35] is a 25-item scale. The scale includes statements about depending on self, having a purpose in life, being positive and true to self, being determined and satisfied with life, taking responsibility for decisions, and having a sense of humor. Each item is scored from 1 (strongly disagree) to 6 (strongly agree). The scale results in a score in the range 25 to 150. High internal consistency has been reported for the scale; Cronbach’s α = 0.94 [37]. The scale achieved the highest score for validity and interpretability of the resilience measures [38].

The Global Transformational Leadership (GTL) Scale [36] was used to measure registered nurses' perception of the transformational leadership style. The 7-item scale measures idealized influence (two items), inspirational motivation (two items), individual consideration (two items), and intellectual stimulation and critical thinking (one item). Each item is scored from 0 (strongly disagree) to 5 (strongly agree).The scale results in a score between 0 to 35. Confirmatory factor analysis has confirmed the scale's construct validity and high internal consistency; Cronbach α = 0.93 [36].

The scale authors permitted translation of the scales into Arabic for the study. A professional fluent in English and Arabic translated the scales and another back translated them independently. A panel of five bilingual registered nurses reviewed the Arabic versions of the scales to ensure readability, clarity, meaningfulness, and face validity. Accordingly, the items were rephrased, synonyms were substituted, and missing words were replaced before conducting the survey [39]. Data collected from those five nurses were not considered part of the final study sample.

### Data collection procedure

Following IRB and hospital director approvals, the questionnaires were delivered with cover letters and consent documents to the hospitals. A graduate assistant, who was not employed at the hospitals, approached the registered nurses and nurse managers to explain the study and distribute the research materials in sealed envelopes. The graduate assistant emphasized the voluntary nature of the survey and the importance of informed consent. The participants were assured of anonymity and confidentiality. The respondents sealed their completed questionnaires in envelopes provided by the graduate assistant and deposited them in a drop box in the office of an assistant administrator at each hospital. The envelopes were collected after 20 days and returned unopened to the first author.

### Statistical analyses

Statistical Product and Services Solutions (SPSS) version 23 was used to analyze the data. All the demographic variables, except the number of dependents, were recoded to create new variables with two groups. Hence, age was recoded into two categories with a cut point at 30 years, marital status was grouped into married and non-married, educational level was grouped as technical versus university, and job designation was re-categorized as bedside nurses versus nurse managers. Similarly, the years of experience variable was recoded with a cut of point at five years, whereas the nursing unit variable was recoded into two subcategories: critical and non-critical. Thirty-six of the nurses (15%) did not fill the survey and were excluded from the analysis; 204 nurses’ responses were considered for analysis.

Descriptive statistics, frequencies and percentages were used to describe the demographic characteristics of the respondents. Internal consistency of the two scales (resilience and GTL) were assessed using the Cronbach’s Alpha. Mean resilience and GTL scores were computed, and 95% confidence intervals were constructed. Statistical significance was set at 0.05. The relationship between resilience and perception of GTL was analyzed using the Pearson correlation. Independent samples t-tests were used to study the relationship between resilience and the demographic variables. ANOVA analysis was performed to compare group means. Variables with a *p*-value less than 0.1 at the univariable level were selected for analysis to allow for the addition of possible confounding variables. Variables were assessed for collinearity and interaction. Finally, selected variables were entered into a stepwise multiple linear regression model to determine the best predictors of resilience.

### Ethical considerations

The study was conducted in accordance with the IRB-approved protocol. The distribution and collection of survey materials ensured the anonymity of the research participants. Confidentiality was maintained by using sealed envelopes to deposit and transport the completed surveys. The data were analyzed on a password-protected computer in a private office at the Hariri School of Nursing. The research records are stored in a locked office and will be destroyed in accordance with the American University of Beirut Archive Policy.

## Results

### Demographic data

Table 1 shows the demographic and work-related data of the 204 subjects, as the response rate was 85%. Approximately two-thirds of the respondents were less than 30 years of age (68%), the majority were females (68%), and half of the respondents were married (48%). Less than half of the respondents (41%) were not responsible for a dependent person. In contrast, a third (32%) were responsible for two or three dependent persons, 20% were responsible for one person, and 7% were responsible for more than four dependents. Concerning educational level, 53% of the respondents had a university degree. Most respondents (85%) were bedside nurses providing direct patient care. More than half (53%) had worked as nurses for less than five years. Approximately 62% had been employed at their hospitals for less than five years. Most of the respondents (55%) worked in critical care units.

**Table 1:** Demographic characteristics of the respondents

Variable	n	%
Age		
Below 30	139	68
Above 30	65	32
Sex		
Females	134	68
Males	62	32
Marital status		
Married	98	48
Non married	105	52
Number of dependents		
None	84	41
1	39	20
2–3	65	32
More than 4	15	7
Educational Level		
Technical	96	47
University	107	53
Designation		
Bedside Nurse	173	85
Nurse manager	30	15
Total years of experience		
Less than five years	108	53
More than five years	96	47
Hospital years of experience		
Less than five years	126	62
More than five years	77	38
Nursing Unit		
Critical	113	55
Non-critical	91	45

### Levels of resilience and perception of global transformational leadership reported by registered nurses working at three hospitals in South Lebanon

Both scales (Resilience and GTL) had excellent internal consistencies with Cronbach's alphas of 0.94 and 0.95, respectively. Resilience scores were in the range of 77–150. The mean resilience score was 119.4 (SD15.3), with 95% confidence interval (117.3–121.5). Perception of Global Transformational Leadership scores were in the range of 0–35, Mean = 25.0 (SD 6.8), at 95% confidence interval (24.0–25.9) (Table 2).

**Table 2:** Descriptive statistics, bivariate correlation, and reliability coefficients for the resilience and GTL scales

Scale	Cronbach's Alpha	mean ± sd	95% CI	Min–Max	Pearson's correlation
**Resilience**	0.94	119.4 ± 15.3	(117.3–121.5)	77–150	0.533*
**GTL**	0.95	25.0 ± 6.8	(24.0–25.9)	0–35	-

^*^ *p* < 0.05

### R*elationship of perception of global transformational leadership scores and resilience scores*

The results showed a significant positive moderate correlation between the perception of global transformational leadership scores and resilience scores among nurses (*p* < 0.01, *r* = 0.533). In addition, there were significant positive moderate to medium correlations between the global transformational leadership and resilience scores. Inspirational motivation scores had a significant positive moderate correlation with the resilience scores (*p* < 0.01, *r *= 0.561). Similarly, the Idealized influence scores had a significant medium correlation with the resilience scores (*p *< 0.01, *r* = 0.485). The Intellectual stimulation score also correlated significantly with the resilience scores (*p* < 0.01, *r* = 0.463). Finally, Individual consideration had a significant medium correlation with the resilience scores (*p* < 0.01, *r* = 0.461).

### Association between demographic and work-related variables, perception of global transformational leadership and resilience score

The unadjusted level t-tests revealed statistically significant associations between resilience and job designation, total years of experience, type of nursing unit, and perception of global transformational leadership scores. Specifically, bedside nurses had significantly lower resilience scores (Mean = 118.3) compared to nurse managers (Mean = 125.4) (*p* = 0.018). Moreover, nurses with less than five years of experience had significantly lower resilience scores (Mean = 117.5) compared to those with more than five years of experience (Mean = 122.3) (*p* = 0.031). In addition, nurses working in critical care areas reported significantly higher resilience scores (Mean = 121.3) compared to non-critical areas (Mean = 117.0) (*p* = 0.049) (Table 3). No significant associations were found between resilience and age, gender, marital status, number of dependents, and educational level.

**Table 3:** Results of independent sample t-test to study associations with resilience scores

Variables	Mean (SD)	*P*-value	T-test
Designation			
Bedside Nurse	118.3 (15.4)	0.018	-2.38
Nurse manager	125.4 (13.6)		
Total years of experience			
Less than five years	117.5 (16.0)	0.031	-2.18
More than five years	122.3 (14.3)		
Nursing Unit			
Critical	121.3 (15.3)	0.049	1.98
Non-critical	117.0 (15.2)		

*Legend*: *SD*  Standard deviation

Variables with a *p*-value less than 0.1 at the univariable level were entered into a forward-stepwise multiple linear regression model to determine the best predictors of resilience. In the multivariable regression, only two predictors significantly contributed to resilience, specifically job designation and perception of global transformational leadership. The results of the regression analysis for designation, total years of experience, nursing unit, and perception of global transformational leadership score are presented in Table 4.

**Table 4:** Linear regression model for independent variables on resilience scores among Registered nurses (*N* = 204)

Variables	B	SE	CI (95%)
Perception of GTL	1.2	0.1	.[0.9,1.4]*
Designation	-5.5	2.6	.[10.5, 0.6]*
Bedside vs. Management			

(Adjusted *R* ^*2*^ = 0.294), *p < 0.05

## Discussion

The study's findings revealed a young workforce, with only a third (32%) of the registered nurses above 30 years of age, which aligns with the Lebanese Order of Nurses’ records [40]. This implies that nurses providing care to patients are relatively inexperienced nurses. These nurses cannot make sound clinical decisions because they lack experience [41]. In addition, inexperienced nurses need help recognizing patient instability and may need more skills to communicate clearly about clinical problems [42]. Therefore, inexperienced nurses lack the competence of their more experienced colleagues [43]. As a result, inexperienced nurses may be less effective in patient care and influence organizational outcomes adversely.

The respondents' mean resilience score was 119.4 (SD 15.3), range 77–150. This score indicates high resilience levels among nurses at the three hospitals. Higher resilience scores have been reported for nurses in South Africa, M = 137.2 (SD 25.7) [44] and nurses in Portugal, M = 138.7 (SD = 18.3) [45]. However, nurses in our study had a higher resilience mean score than Greek nurses, M = 76.69 (SD = 16.22) [22], Iranian nurses, M = 63.8, (16.2) [46]. and, Singaporean nurses M = 25.9, (SD = 6) [47]. Moreover, our study’s nurses’ resilience scores were higher than those reported in a recent national study during the pandemic (M = 72, SD = 13.5) [48].

The mean score for perceived global transformational leadership was 25.0 (SD 6.8), range 0–35, indicating that the nurses perceived their leaders as transformational. A moderately significant correlation was noted between the perception of global transformational leadership scores and the nurses' resilience scores. Our results imply that the nurses’ perceptions of global transformational leadership may partly explain why they had higher resilience scores than those reported in the studies cited in the previous paragraph. However, the moderate linear relationship between the nurses’ resilience acores and their global transformational leadership, *r* = 0.533, explains less than 30% of the variance in the measures. Factors other than perceptions of global transformational leadership influence nurses’ resilience. Our finding that nurses’ designation is a significant factor in explaining nurses’ resilience is consistent with other findings such as Sull et al.’s (2015) that showed clinical staff had less resilience than staff with line management responsibilities [20]. Furthermore, Hsieh et al. (2016) reported a positive correlation between resilience and seniority among Taiwanese nurses [49]. Ang et al. (2018) also found a significant positive relationship between nurses in administrative roles and resilience in Singapore [47]. Lower resilience levels among bedside nurses is related to constant pressure in the clinical setting caused by increase in patient acuity and complexity, reduced resources, workload, lack of communication and support, workplace insecurity [50].

Studies suggest that working in a management role fosters resilience because the nurses in these positions have better life-work balance compared to bedside nurses [51]. Furthermore, organizational support [52], disaster experiences [53] and coping with the stressors in nurse managers' roles helps to build resilience [54]. Dealing with competing priorities also has a positive effect on nurse managers' resilience [55]. Moreover, observing the positive impact of decisions taken as a nurse manager on patients can explain the higher resilience of nurses in non-clinical roles [56].

The significant positive linear relationship between higher resilience scores and years of experience we reported is consistent with other studies [47, 57]. However, Sull et al. (2015) and Hsieh et al. (2017) [20, 49] found no significant association between years of experience and resilience. Demographic differences might explain differences between our results and those of Sull et al. (2015). Whereas we collected data from registered nurses, Sull et al. included a diverse range of non-nurse health workers in their study. Moreover, Hsieh et al.’s sample included only nurses who had experienced abuse. Consequently, the sample difference of Sull et al. and Hsieh et al. may explain the contradictory findings with ours.

Mealer et al. (2012) reported that younger intensive care nurses with less nursing experience have higher resilience scores than their older and more experienced colleagues. The small but significant difference is probably because the more experienced nurses have had more time to accumulate the stressors of working in an intensive care unit [58]. In this study results revealed a significant relationship between nursing unit and resilience (*p* = 0.049) whereby nurses working in critical areas declared more resilience than those working in non-critical areas. Monomenidis et al. (2019) reported similar findings from their study of Greek nurses [22].

Our multivariate linear regression model results indicate that being a bedside nurse resulted in a 5.5 point decrease in resilience score compared to nurses in management positions (B = -5.5, *p* = 0.029). In addition, the regression model indicates that every one unit increase in the perception of global transformational leadership scores is associated with a 1.2 points increase in resilience score (B = 1.2, *p* < 0.01). The association between global transformational leadership perception scores and resilience scores might be because by modelling, enthusiasm, and status, nurse managers encourage higher levels of work engagement [59]. Moreover, idealized influence is expected to produce emotional arousal among followers displayed by dedication and courage [60]. Empowerment enhances employees' self-efficacy and boosts their work satisfaction, resulting in organizational commitment [18]. Similarly, intellectual stimulation fosters followers' perspectives-taking capacity, open-mindedness, creativity, and love of learning, reflecting positive character strength [61].

### Directions for future research

Studying components of resilience such as hope and optimism, job designation, and perception of global transformational leadership will help achieve a better predictive model. Replicating this study using a stratified sampling design at a national level across all hospitals is needed to explore predictors of resilience across government and private, rural and urban, and teaching and non-teaching hospitals to generalize results. The results of a Lebanon-wide study could contribute to a national agenda for increasing resilience among bedside nurses and, possibly, decreasing Lebanon’s nursing shortage, especially in Sidon and other underserved areas.

### Implications for National Health Policy

National nursing workforce policies are required that adopt a comprehensive strategy for strengthening the nursing workforce in underserved areas. A pyramid approach that considers individual, organizational, health system level, and institutional factors offer a better prospect of success than interventions that target individuals in isolation [62, 63]. Authorizing advanced nursing practice to enable nurses in underserved areas to work to their full potential would be an essential reform. Experience in delivering health services in underserved areas has shown that nurses with advanced competencies are more resilient to challenges and able to provide a high quality of care for vulnerable populations [64].

### Implications for nurse administrators

Hospital administrations in Lebanon should implement organizational strategies to foster transformational leadership and strengthen resilience among bedside nurses. Such strategies include training, self-development programs, mentoring to improve workplace relationships and teamwork, and promoting work-life balance and spirituality to strengthen nurses’ coping strategies and improve nurse retention. Team-based approaches to workforce development are preferred because they can foster a shared sense of meaning and purposefulness [65]. Nurses in management and supervisory positions in underserved areas should be prioritized for intervention because their advocacy is essential for urging hospital administrations to lobby the government for resources to improve work environments and practice protocols [66].

### Limitations

The study has several limitations. The major limitation is that it relies on a self-reported measure of resilience, resulting in possible information bias. Another limitation is that the reported results might have been influenced by factors affecting the hospitals at the time of the survey. Such factors might include political and economic tensions affecting the region and its hospitals. A third limitation is possible convenience sample bias. Furthermore, the results reported cannot be generalized beyond the hospitals where the study was conducted.

## Conclusions

The study's primary findings were that job designation and perceptions of global transformational leadership predict resilience among nurses in Sidon and adjacent underserved areas. Moreover, job designation, years of experience, clinical specialty, and perception of global transformational leadership were significantly associated with resilience. These findings can encourage hospital administrations and nurse leaders to implement strategies to strengthen nurses’ resilience in Sidon and other underserved and resource-poor areas in Lebanon. The sustainability of the nursing workforce in underserved areas of Lebanon depends on interventions to build resilience and improve leadership. National, regional, and international studies are required to determine whether the reported findings can be generalized. Meanwhile, nurse managers and supervisors can strengthen nurses’ resilience, contribute to retention, and improve patient outcomes by adopting a transformational leadership style. The Order of Nurses might play a major role in training nurse managers in transformational leadership not only in that region but also across the country. Nonetheless, policy reforms are needed to develop and sustain the nursing workforce in underserviced areas of Lebanon.

## Data Availability

The study dataset is available from the corresponding author upon reasonable request.
